# Neighborhood Disadvantage, Neighborhood Safety and Cardiometabolic Risk Factors in African Americans: Biosocial Associations in the Jackson Heart Study

**DOI:** 10.1371/journal.pone.0063254

**Published:** 2013-05-14

**Authors:** Cheryl R. Clark, Mark J. Ommerborn, DeMarc A. Hickson, Kya N. Grooms, Mario Sims, Herman A. Taylor, Michelle A. Albert

**Affiliations:** 1 Division of General Medicine and Primary Care, Brigham and Women’s-Faulkner Hospitalist Program, Boston, Massachusetts, United States of America; 2 Center for Community Health and Health Equity, Brigham and Women’s Hospital, Boston, Massachusetts, United States of America; 3 Jackson Heart Study, Jackson State University, Jackson, Mississippi, United States of America; 4 Department of Medicine, University of Mississippi Medical Center, Jackson, Mississippi, United States of America; 5 Division of Cardiovascular Medicine, Howard University, Washington, D.C., United States of America; NIDDK/NIH, United States of America

## Abstract

**Objective:**

We examined associations between neighborhood socioeconomic disadvantage, perceived neighborhood safety and cardiometabolic risk factors, adjusting for health behaviors and socioeconomic status (SES) among African Americans.

**Methods:**

Study participants were non-diabetic African Americans (n = 3,909) in the baseline examination (2000–2004) of the Jackson Heart Study. We measured eight risk factors: the metabolic syndrome, its five components, insulin resistance and cardiovascular inflammation. We assessed neighborhood socioeconomic disadvantage with US Census 2000 data. We assessed perceived neighborhood safety, health behaviors and SES via survey. We used generalized estimating equations to estimate associations with a random intercept model for neighborhood effects.

**Results:**

After adjustment for health behaviors and SES, neighborhood socioeconomic disadvantage was associated with the metabolic syndrome in women (PR 1.13, 95% CI 1.01, 1.27). Lack of perceived safety was associated with elevated glucose (OR 1.36, 95% CI 1.03, 1.80) and waist circumference (PR 1.06, 95% CI 1.02, 1.11) among women, and with elevated glucose (PR 1.30, 95% CI 1.02, 1.66) and insulin resistance (PR 1.25, 95% CI 1.08, 1.46) among men.

**Conclusions:**

Neighborhood socioeconomic disadvantage and perceived safety should be considered as targets for intervention to reduce cardiometabolic risks among African Americans.

## Introduction

Cardiometabolic risk factors, including abdominal obesity, elevated blood pressure, dyslipidemia, glucose intolerance and inflammation are highly prevalent among African Americans [1]–[3]. Adults with multiple cardiometabolic risk factors who meet criteria for the metabolic syndrome have an increased risk for cardiovascular disease (CVD) events [4], [5]. National epidemiologic surveillance data suggest that health behaviors such as physical inactivity and diet strongly contribute to the high prevalence of cardiometabolic risk factors seen among African Americans [6].

Increasingly, the residential neighborhood socioeconomic environments to which African Americans are exposed are hypothesized to contribute to cardiometabolic risks in these groups [7]. Neighborhood socioeconomic environments are thought to influence health behaviors through material resources such as access to healthy foods, and through social norms around exercise and smoking [8]–[10]. However, the extent to which neighborhood socioeconomic environments have *independent* associations with cardiometabolic risk factors, above and beyond associations with health behaviors, has not been fully explored in African Americans [11]. African Americans in the United States (US) are likely to live in residential environments with high levels of health-related *neighborhood socioeconomic disadvantages*, including high unemployment and poverty, as well as high levels of *perceived social disorder*, such as lack of perceived neighborhood safety [12], [13]. Neighborhood socioeconomic disadvantage and disorder are theorized to contribute to psychological stress which activates the hypothalamic-pituitary-adrenal (HPA) axis, which may lead to physiologic dysfunction in multiple pathways, including cardiovascular inflammation and metabolic derangements [14], [15].

Recent population-based studies have shown independent associations between neighborhood disadvantage and cardiometabolic risks. The Atherosclerosis Risk in Communities study found that low neighborhood socioeconomic status (SES) was associated with an increased prevalence of the metabolic syndrome among African American women in the southeastern US, but not among African American men, a finding that was not completely explained by alcohol use, smoking, or physical activity among women [16]. It is also possible that neighborhood socioeconomic disadvantage and perceived disorder (e.g., perceived neighborhood safety) may be differentially associated with some cardiometabolic risk factors more so than others. As an example, Laraia et al. found that among diabetic patients, low neighborhood SES was more closely associated with obesity and glucose control than measures of blood pressure and dyslipidemia [17].

To date, associations between neighborhood socioeconomic disadvantage, perceived disorder and specific cardiometabolic risk factors have yet to be fully explored among African Americans. The 2004 National Heart, Lung and Blood Institute/American Heart Disease Association conference on the definition of metabolic syndrome described specific etiologic cardiometabolic factors that may underlie the pathogenesis of the metabolic syndrome: (1) abdominal fat around the waist, (2) insulin resistance, and (3) other mechanisms such as cardiovascular inflammation [18]. A better understanding of which underlying metabolic factors and physiologic mechanisms are connected to neighborhood socioeconomic disadvantage and perceived disorder may assist in identifying additional populations at risk for cardiometabolic diseases earlier in the course of the development of cardiovascular disease, and may improve population-level prevention efforts by suggesting specific factors to track and address among those exposed to neighborhood socioeconomic disadvantage and perceived disorder.

Thus, our study examined cross-sectional associations between features of neighborhood socioeconomic disadvantage, health behaviors, and cardiometabolic risk factors among African American participants in the Jackson Heart Study (JHS) who reside in the Jackson, Mississippi Metropolitan Statistical Area (MSA), a geographic area with a high prevalence of cardiovascular risk factors. Specifically, we examined whether socioeconomic aspects of residential neighborhoods (neighborhood socioeconomic disadvantage, perceived neighborhood safety) are associated with health behaviors (dietary intake, physical activity, smoking), cardiometabolic risk factors (the metabolic syndrome, and its component characteristics) and biomarkers associated with abdominal obesity, inflammation and insulin resistance. We hypothesized that neighborhood socioeconomic disadvantage and perceived neighborhood safety would have independent associations with the metabolic syndrome and its constituent components, above and beyond associations with health behaviors. We additionally hypothesized that bio-measures of three etiologic factors, abdominal adiposity, cardiovascular inflammation and insulin resistance were associated with neighborhood socioeconomic disadvantage and perceived neighborhood safety. We examined sex differences in the above associations.

## Methods

### Study Design and Population Sample

The JHS is an observational cohort study designed to investigate factors that influence the development and worsening of CVD in African Americans [19]. Participants of the JHS are adult, non-institutionalized residents of three counties (Hines, Madison, Rankin counties) that comprise the Jackson MSA. JHS participants are geographically representative of the distribution of the African American population within the Jackson MSA [20].

Methods describing participant recruitment and sample characteristics have been reported previously [21]. Between 2000 and 2004, African American men and women aged 35 to 84 were recruited from 4 sources: (1) the Jackson, Mississippi site of the ARIC study, a probability sample from the Jackson City driver’s license registry; (2) a random sample of all residents of the tri-county area aged 35 to 84 obtained from a commercially available list (Accudata America); (3) a volunteer sample of tri-county area residents who met age and demographic criteria; and (4) first-degree relatives of participants in each of the ARIC, random, and volunteer samples. First degree relatives aged 21 and older were eligible to participate. The complete JHS cohort includes 5,301 African American men and women aged 21 to 95 years of age. The present analysis estimates cross-sectional associations among JHS participants who were not diabetic at the baseline assessment and who therefore did not provide fasting blood samples at baseline. The JHS study protocol was approved by the Institutional Review Boards of Jackson State University, Tougaloo College and the University of Mississippi Medical Center, and all participants provided written informed consent. These analyses were approved by the Partners HealthCare Institutional Review Board.

### Measures

#### Cardiometabolic risk factors

We examined eight measures of cardiometabolic risk: the metabolic syndrome, the five constituent components of the metabolic syndrome (elevated serum triglycerides, fasting plasma glucose, blood pressure, waist circumference, and decreased high density lipoprotein cholesterol [HDL-C]); a measure of cardiovascular inflammation, c-reactive protein (CRP); and a measure of insulin resistance, as defined by the homeostasis model assessment of insulin resistance (HOMA-IR) [22]. We defined metabolic syndrome and its constituent components among non-diabetics, consistent with criteria from the National Cholesterol Education Program’s Adult Treatment Panel (ATP) III guidelines [18], [23]. Participants were defined as having the metabolic syndrome if they had at least 3 of 5 of the following characteristics: (1) a fasting serum triglyceride level greater than 150 mg/dL or the use of anti-lipid (fibrate or nicotinic acid derivative) medications; (2) fasting plasma glucose between 100 mg/dL and 125 mg/dL; (3) systolic blood pressure greater than 130 mmHg, elevated diastolic blood pressure greater than 85 mmHg, a self-reported physician’s diagnosis of hypertension, or the use of anti-hypertensive agents; (4) fasting serum HDL-C less than 50 mg/dL for women, less than 40 mg/dL for men, or the use of anti-cholesterol medications; and (5) waist circumference greater than 88 cm in women, and greater than 102 cm in men. C-reactive protein was assessed as a physiologic measure of cardiovascular inflammation, separate from the criteria for the metabolic syndrome. C-reactive protein was defined as elevated at a level of 3.0 mg/L or greater. HOMA-IR was calculated according to Matthews et al. as follows: fasting plasma glucose (mmol/L) × fasting insulin (uU/ml)/22.5); an elevated level of insulin resistance was defined at a value of 3.31 or higher, consistent with HOMA-IR levels predicting progression to diabetes in an African American cohort as detailed by Osei et al [24].

Eight hour fasting triglyceride, HDL cholesterol, insulin and glucose levels were obtained via venipuncture. Assays and procedures for measuring fasting whole blood, plasma and serum-derived analytes in the JHS have been previously described [25]. The use of anti-lipid, anti-glycemic, and anti-cholesterol medications within two weeks prior to the baseline clinic exam were assessed via survey data. C-reactive protein was measured with a high-sensitivity immunoturbidimetric CRP-Latex assay (Kamiya Biomedical Company).

#### Neighborhood socioeconomic disadvantage

We defined neighborhoods at the census tract level using the 2000 US Census (Census 2000) boundaries. We used the theory of Social Disorganization to select measures of neighborhood socioeconomic conditions, in order to create an index of neighborhood socioeconomic disadvantage in Jackson MSA neighborhoods [26]. Ten measures obtained from Census 2000 were used to measure themes in Social Disorganization Theory that relate to neighborhood socioeconomic conditions: *socioeconomic disadvantage* (percentage of adults below poverty in the neighborhood, the civilian unemployment rate, percentage of college educated neighborhood residents, percentage of households with no vehicle ownership); *racial heterogeneity and family structure* (percentage of the population that is black or African American, percentage of female headed households in the neighborhood, percentage of the population aged 15 to 24); *urbanicity* (percentage of neighborhoods in urban areas) and *residential stability* (percentage of resident who lived in the same neighborhood five years prior, percentage of owner occupied housing). We used exploratory factor analysis with an oblique PROMAX rotation to construct an index of *neighborhood socioeconomic disadvantage* from these ten candidate measures. An a priori factor loading (standardized regression coefficient) of 0.50 was used to determine whether a covariate loaded on any extracted factor. The reliability of the neighborhood socioeconomic disadvantage index was assessed via standardized Cronbach’s alpha coefficients.

#### Perceived neighborhood safety

JHS survey data assessed neighborhood safety perceptions among individual participants with a single item statement “this neighborhood is safe from crime.” Data on perceived safety were collected during an annual follow-up call conducted approximately on the third anniversary of their clinic visit date. Participants who agreed or strongly agreed were categorized as perceiving their neighborhood as safe; those who disagreed or strongly disagreed were categorized as perceiving their neighborhood as unsafe.

#### Covariates

We used self-reported measures of covariates related to cardiometabolic risk factors, and exposure to neighborhood socioeconomic disadvantage including *health behaviors* (physical activity, smoking status, dietary intake), the *individual’s socioeconomic position* (family income and educational level), as well as the participant’s age and sex. We measured physical activity among JHS participants with a validated *active living index*, which is a summary score of the (1) frequency and duration of activities performed during leisure-time, and lifestyle activities including walking and biking during errands, and while commuting to and from work, (2) minus the frequency of television watching as an indicator of sedentary behavior [27]. Smoking status was categorized as current, former, and never smoked. Dietary intake was assessed by a well-validated food frequency questionnaire adapted for adults living in the Mississippi Delta Region [28], [29]. In this analysis, we assessed participants’ average daily intakes of total energy (kcal/day), total fiber (g/day), percentage of daily calories consumed from alcohol, percentage of calories from protein, and we identified participants who met United States Department of Agriculture [30] recommendations on the proportion of the participant’s macronutrient intake that came from carbohydrates and fats. Annual family income was scaled for family size [31], [32]. Educational attainment was categorized as less than high school graduation, high school graduate, some college, and college graduate.

### Statistical Analysis

#### Individual-level data

Consistent with ATP III guidelines, we assessed cardiometabolic risk factors for the metabolic syndrome among non-diabetic patients. We excluded 1,236 (23%) participants with diabetes, defined as fasting plasma glucose ≥126 mg/dL, a self-reported physician’s diagnosis of diabetes, or self-reported use of anti-glycemic medications [1]. We also excluded 15 participants with implausible energy intakes less than or equal to 400 calories daily [8]. The largest source of missing data due to item non-response was on participant family income or family size, missing in 866 JHS participants (16%). Additionally, the baseline questionnaire assessing neighborhood safety was not administered to 937 JHS participants (18%). To reduce bias that might have resulted from list-wise deletion, chiefly, bias due to reduced power from missing data, we used multiple imputation with the PROC MI and PROC MIANALYZE procedures in SAS® [33]. In multivariable models, we imputed missing values employing the Markov Chain Monte Carlo (MCMC) method in PROC MI, to use all observed dependent and independent covariate values in the model to impute values for missing covariate data [33], [34]. Missing data were imputed for family income and perceived neighborhood safety, as well as for other missing outcomes and covariates (cardiometabolic risk factors, health behaviors, sociodemographic covariates) which had minimal missing data for each variable (between 0.1 percent and 6.1 percent of the analytic sample). There were no statistically significant differences between the metabolic syndrome and missing data on income, perceived safety, or other health behaviors, and sociodemographic covariates.

After the above exclusions, in multivariable models, we additionally excluded 51 individuals whose residential addresses could not be assigned to one of the 102 JHS neighborhoods (census tracts). We also excluded individuals living in census tracts with very few numbers of JHS participants per neighborhood (fewer than 10), resulting in the additional exclusion of 90 JHS participants and 21 (21%) Jackson MSA neighborhoods [35]. After exclusions and multiple imputation of missing data, the final analytic JHS cohort was N = 3,909 participants.

Descriptive statistics at the individual level were performed with Chi-squared tests of associations among proportions, and Kruskal-Wallis non-parametric tests for associations between categorical and continuous variables.

#### Neighborhood-level data and multi-level models

To estimate associations between each cardiometabolic risk factor, neighborhood-level condition, and individual-level covariate, we estimated prevalence ratios (PR) using log binomial regression, and odds ratios (OR) using binomial regression with a logit link, within a generalized estimating equations framework in the PROC GENMOD procedure in SAS® [36]. For common cardiometabolic risk factors, prevalence ratios were estimated. For rare outcomes where a prevalence ratio could not be estimated, the odds ratio was obtained [36]. To account for the multi-level structure of the data we fit a random intercept model for each neighborhood within the GENMOD procedure. To conserve power, statistically non-significant individual-level and neighborhood-level variables were removed from the final models. All descriptive statistics and models were analyzed in SAS® version 9.2 (SAS® Institute, Cary, NC).

## Results

### Measuring Neighborhood Socioeconomic Disadvantage

The exploratory factor analysis identified four covariates that “loaded” on, or were tightly associated with, the first extracted factor which we identified as *neighborhood socioeconomic disadvantage*: the percentage of adults living in poverty (factor loading 0.82), the civilian unemployment rate (factor loading 0.77), the percentage of households with no car ownership (factor loading 0.97), and the percentage of owner occupied housing in the neighborhood (factor loading −0.55). The constructed index, constructed as the sum of all four covariates weighted by their factor loadings, had poor reliability (Cronbach’s alpha = 0.28). Thus, the *neighborhood socioeconomic disadvantage* (NSED) index was re-constructed as the weighted sum of the three covariates with the highest factor loadings (adults in poverty, civilian unemployment rate, no car ownership). The resultant index had excellent reliability (Cronbach’s alpha = 0.94).

The NSED index ranged from a minimum value of 0.82 (low disadvantage) to a maximum of 97.8 (high disadvantage) with a median value of 18.1 and standard error of 2.0. Table S1 shows the ecological characteristics of Census 2000 participants in Jackson MSA neighborhoods that were above (most advantaged neighborhoods) and below the median NSED score (most disadvantaged neighborhoods). The division of most advantaged and most disadvantaged neighborhoods based on the median NSED score had excellent convergent validity with other neighborhood characteristics, as predicted by the theory of Social Disorganization. Compared to the most advantaged neighborhoods, the most disadvantaged neighborhoods had a lower percentage of college educated adults (13% vs. 37%), a lower percentage of owner occupied housing (57% vs. 84%), and a significantly higher black population (83% vs. 18%) in the Census 2000 population (Table S1). However, *neighborhood stability* (percentage of neighbors in place since 1995) was not associated with neighborhood disadvantage (57 vs. 52%, p = 0.38); this measure was included as a separate covariate in subsequent multivariable models.

### Participant Characteristics and Associations with Cardiometabolic Risk Factors

Table 1 shows the distribution of JHS participants in the most advantaged and disadvantaged neighborhoods. JHS participants were much more likely to live in the most disadvantaged neighborhoods than the most advantaged neighborhoods (73% vs. 27%). Compared to JHS participants in the most advantaged neighborhoods, JHS participants in the most disadvantaged neighborhoods were older (aged 55 vs. 50 years), had lower median family household incomes ($25,096 vs. $29,836), were less likely to have completed a college bachelor’s degree (33% vs. 36%), were less active (active living index 2.0 vs. 2.3), and were more likely to perceive their neighborhoods as unsafe (41% vs. 36%). Dietary intake was not substantively different between those in the most advantaged and disadvantaged neighborhoods. Those in the most advantaged neighborhoods were slightly more likely to meet USDA recommendations for carbohydrate intake (95% vs. 93%), and were more likely to have high protein intake (39% vs. 35%) than those living the most disadvantaged neighborhoods. The percentage of total calories consumed from alcohol was small (1.3% vs. 1.2%) in both groups.

**Table 1:** Neighborhood Socioeconomic Disadvantage and Selected Characteristics of Jackson Heart Study Participants, 2000–2004.

	All Neighborhoods	Most Advantaged Neighborhoods	Most Disadvantaged Neighborhoods	P Value
**JHS Demographic and Social Characteristics**	3909	1057 (27)	2852 (73)	-
Age, median (IQR), y	53 (44, 63)	50 (43, 60)	55 (44, 64)	<0.0001
Female sex	2454 (63)	673 (64)	1781 (62)	0.48
JHS median family income, median (IQR), $	27,995 (14,000–47,468)	29,836 (14,821–47,747)	25,096 (14,000–44,290)	0.02
Educational attainment				<0.01
*< High school*	625 (16)	136 (13)	489 (17)	
* High school graduate*	782 (20)	200 (19)	582 (20)	
* Some college*	1154 (30)	334 (32)	820 (29)	
* Completed bachelor degree or greater*	1334 (34)	384 (36)	950 (33)	
Perceives neighborhood as unsafe	1310 (40)	331 (36)	979 (41)	<0.01
**JHS Behavioral Characteristics**				
Active Living Index, median (IQR)	2.0 (1.5, 2.8)	2.3 (1.5, 2.8)	2.0 (1.3, 2.8)	0.05
Smoking status				0.10
* Never smoked*	2675 (69)	744 (71)	1931 (68)	
* Former smoker (>100 cigarettes)*	669 (17)	159 (15)	510 (18)	
* Current smoker*	533 (14)	147 (14)	386 (14)	
Daily caloric energy intake, median (IQR), kcal	1955 (1412, 2737)	1975 (1426, 2773)	1951 (1406, 2718)	0.29
Meets USDA recommended percentage ofcalories as carbohydrate^a^	3651 (93)	1002 (95)	2649 (93)	0.03
Meets USDA recommended percentage ofcalories as fat^b^	1970 (50)	507 (48)	1463 (51)	0.06
Average percentage of calories from alcohol, %	1.2	1.3	1.2	0.41
≥15% of calories from protein	1378 (36)	406 (39)	972 (35)	0.01
Total dietary fiber intake, median (IQR), g	15 (11, 19)	14 (11, 19)	15 (11, 19)	0.75

Numbers are N(%) unless otherwise noted. Chi-squared tests for associations among categorical values. Kruskal-wallis statistic used to test for associations between classes for continuous outcome variables.

a USDA recommended percentage of calories as carbohydrates is ≤65%.

b USDA recommended percentage of calories as fat is ≤35%.

There were no sex differences in living in the most advantaged and disadvantaged neighborhoods. There were sex differences in median family household income and perceived neighborhood safety. Compared to men, women had lower median household incomes ($23,053 vs. $32,658, p<0.0001), and were more likely to perceive their neighborhoods as unsafe (42% vs. 37%, p<0.01) [data not shown in tables].

**Figure 1:**
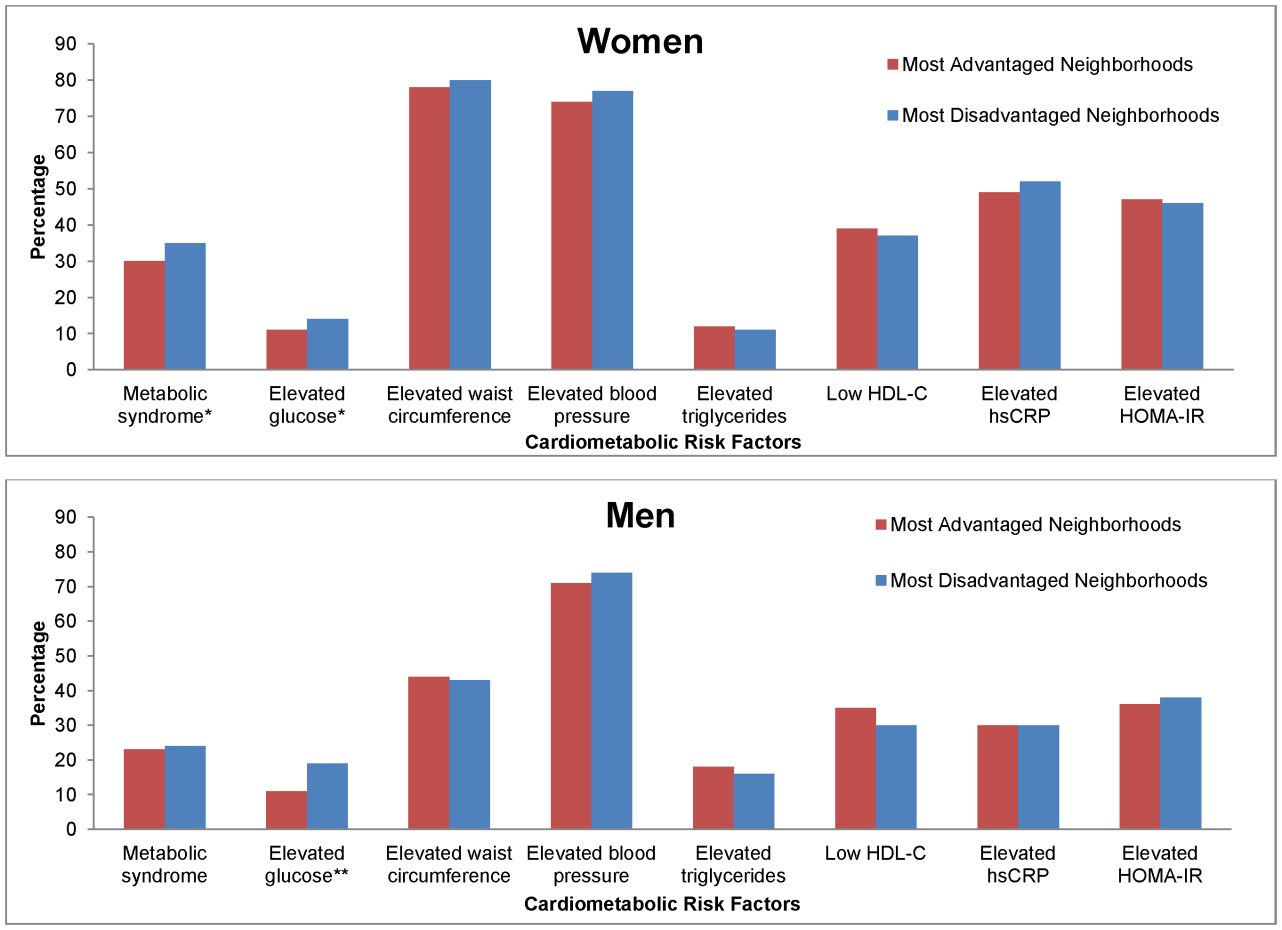
Unadjusted Percentage of Cardiometabolic Risk Factors between Neighborhood Socioeconomic Disadvantage among Women and Men in the Jackson Heart Study, 2000–2004. Excludes diabetics consistent with ATP III consensus guidelines and individuals with ≤400 kcal daily energy intake. Diabetes is defined as self-reported type I or II diabetes; taking diabetes medications; having a measured fasting plasma glucose equal to or greater than 126 mg/dL; measured hemoglobin A1C of 6.5% or greater. Elevated glucose (“pre-diabetes”) is defined as a measured fasting plasma glucose between 100–125 mg/dL, consistent with American Diabetes Association recommendations. Sex specific norms are used to define elevated waist circumference and low HDL measurement. Elevated blood pressure is defined as systolic blood pressure ≥130 mmHg or diastolic blood pressure ≥85 mmHg. Elevated triglycerides are defined as ≥150 mg/dL. Elevated hsCRP is defined as hsCRP≥3.0 (mg/L), elevated insulin resistance is defined as HOMA-IR greater than or equal to 3.31. *p≤0.05 **p≤0.01.

**Figure 2:**
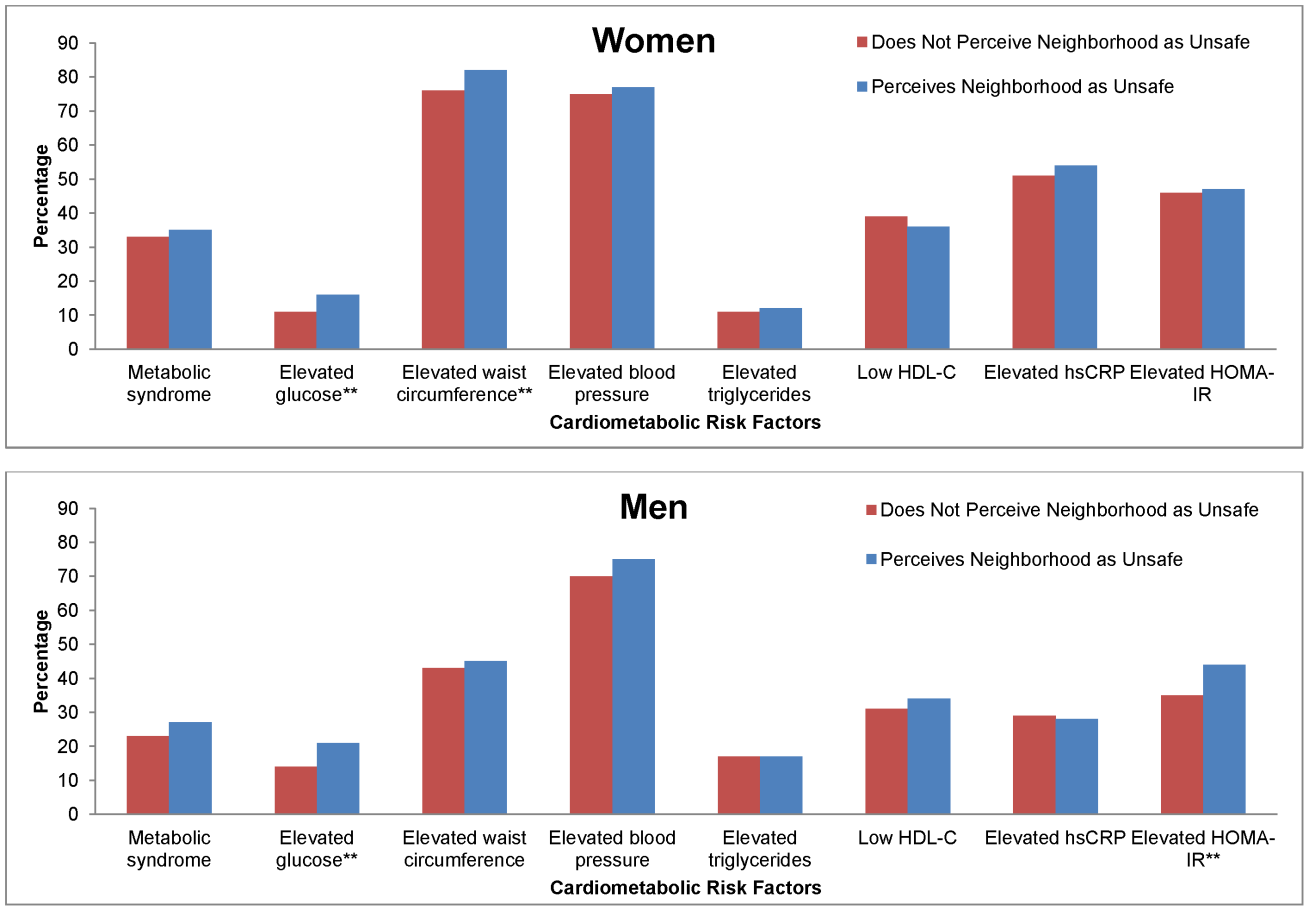
Unadjusted Percentage of Cardiometabolic Risk Factors between Neighborhood Safety among Women and Men in the Jackson Heart Study, 2000–2004. Excludes diabetics consistent with ATP III consensus guidelines and individuals with ≤400 kcal daily energy intake. Diabetes is defined as self-reported type I or II diabetes; taking diabetes medications; having a measured fasting plasma glucose equal to or greater than 126 mg/dL; measured hemoglobin A1C of 6.5% or greater. Elevated glucose (“pre-diabetes”) is defined as a measured fasting plasma glucose between 100–125 mg/dL, consistent with American Diabetes Association recommendations. Sex specific norms are used to define elevated waist circumference and low HDL measurement. Elevated blood pressure is defined as systolic blood pressure ≥130 mmHg or diastolic blood pressure ≥85 mmHg. Elevated triglycerides are defined as ≥150 mg/dL. Elevated hsCRP is defined as hsCRP ≥3.0 (mg/L), elevated insulin resistance is defined as HOMA-IR greater than or equal to 3.31. **p≤0.01.

Figure 1 shows the prevalence of metabolic syndrome, its constituent components, elevated CRP as a marker of inflammation, and HOMA-IR as a physiologic measure of insulin resistance, in the most advantaged and disadvantaged neighborhoods by sex. Among women, both elevated waist circumference and blood pressure were the most prevalent components of the metabolic syndrome. Women in the most disadvantaged neighborhoods had a higher prevalence of the metabolic syndrome (35% vs. 30% p = 0.01) and elevated fasting glucose levels (14% vs. 11%, p = 0.03) than those in the most advantaged neighborhoods. Among men, those in the most disadvantaged neighborhoods had a higher prevalence of elevated glucose (19% vs. 11%, p = 0.01), and a *lower* prevalence of low HDL-C (30% vs. 35%, p = 0.05) compared to men in the most advantaged neighborhoods. Neighborhood socioeconomic disadvantage was not associated with either CRP or HOMA-IR in men or women. Figure 2 shows associations between cardiometabolic risk factors and perceptions of neighborhood safety. Among women, perceiving the neighborhood as unsafe was associated with having an elevated fasting glucose level (16% vs 11% p<0.01), and elevated waist circumference (82% vs 76% p<0.01). Among men, perceiving the neighborhood as unsafe was associated with an elevated fasting glucose (21% vs. 14%, p<0.01) and an elevated HOMA-IR (44% vs. 35%, p = 0.01).

### Multivariable Associations among Cardiometabolic Risk Factors, Neighborhood Socioeconomic Disadvantage, Perceived Neighborhood Safety, and Covariates

Table 2 shows models of the adjusted associations between the metabolic syndrome, neighborhood socioeconomic disadvantage (lives in the most disadvantaged neighborhoods vs. most advantaged neighborhoods), and perceived neighborhood safety (perceives the neighborhood as unsafe vs. does not perceive lack of safety) by sex. After adjustment for age, there was an increased prevalence of the metabolic syndrome among women living in the most disadvantaged neighborhoods compared to those in the most advantaged neighborhoods (PR 1.13, 95% Confidence Interval (CI) 0.99, 1.28). After adjustment for age, perceived neighborhood safety, and health behaviors, a statistically significant association was seen between neighborhood socioeconomic disadvantage and metabolic syndrome among women (PR 1.13, 95% CI 1.01, 1.27). The effect estimate of the association between neighborhood socioeconomic disadvantage and metabolic syndrome remained unchanged after further adjustment for neighborhood stability, health behaviors and individual-level SES (PR 1.13, 95% CI 1.01, 1.27). Active living as a measure of physical activity (PR 0.93, 95% CI 0.87, 0.99), smoking (PR 1.51, 95% CI 1.32, 1.74), and neighborhood stability (PR 0.93, 95% CI 0.87, 0.98) were associated with the metabolic syndrome in women (data not shown in tables).

**Table 2:** Correlates of Metabolic Syndrome in the Jackson Heart Study Prevalence Ratios and (95% Confidence Intervals).

	Adjusted for Age	Adjusted for Age and Health Behaviors^a^	Adjusted for All Covariates^b^
**Women**			
**N = 2,454**			
Lives in most disadvantaged neighborhoods	1.13 (0.99, 1.28)	1.13 (1.01, 1.27)	1.13 (1.01, 1.27)
Perceives neighborhood as unsafe	1.02 (0.89, 1.16)	1.00 (0.88, 1.14)	1.00 (0.88, 1.14)
**Men**			
**N = 1,455**			
Lives in most disadvantaged neighborhoods	1.00 (0.80, 1.25)	0.97 (0.79, 1.18)	0.96 (0.79, 1.18)
Perceives neighborhood as unsafe	1.15 (0.97, 1.37)	1.14 (0.96, 1.36)	1.16 (0.98, 1.38)

Figures estimate age-adjusted associations with metabolic syndrome via binomial log regression with multiple imputations for missing covariates performed in PROC GENMOD, with repeated statement for neighborhood census tracts. Reference categories: Lives in most advantaged neighborhoods, does not perceives neighborhood as unsafe. Analysis for metabolic syndrome excludes all diabetics (self-reported, use of diabetic medications, elevated fasting plasma glucose equal or greater than 126 mg/dL, or hemoglobin A1C greater or equal to 6.5%) and individuals with ≤400 kcal daily energy intake.

a Models estimate the prevalence ratios associated with neighborhood socioeconomic disadvantage and perceived neighborhood safety adjusted for active living index, current smoking status, meeting USDA diet recommendations for percentage of calories as carbohydrate, fat, greater than 15% of daily calories as protein, daily dietary fiber intake, and percentages of calories as alcohol.

b Adjusts for age, health behaviors, lives in most disadvantaged neighborhoods, perceives neighborhood as unsafe, neighborhood stability, family household income scaled for family size, and less than high school educational attainment.

Perceived neighborhood safety was not associated with metabolic syndrome among women in any of the sequential multivariable models. Neither neighborhood socioeconomic disadvantage nor perceived neighborhood safety were associated with the metabolic syndrome in men (Table 2).

Table 3 shows adjusted associations between elevated glucose, neighborhood socioeconomic disadvantage, and perceived neighborhood safety by sex. Among women, the rare prevalence of elevated glucose necessitated estimating associations with the odds ratio measure. In fully adjusted models, elevated glucose was associated with perceived safety among both women (OR 1.36, 95% CI 1.03, 1.80), and men (PR 1.30, 95% CI 1.02, 1.66). Neither health behaviors or socioeconomic status fully attenuated the associations between perceived neighborhood safety and elevated glucose among women or men. However, neighborhood socioeconomic disadvantage was not associated with elevated glucose among women or men (Table 3). Table 4 shows adjusted associations between neighborhood socioeconomic disadvantage, perceived neighborhood safety, and biomeasures of physiologic processes thought to underlie the development of metabolic syndrome and abnormal glucose metabolism: (1) insulin resistance (HOMA-IR), (2) abdominal adiposity (elevated waist circumference), and (3) cardiovascular inflammation (CRP). Among women, neighborhood socioeconomic disadvantage was not associated with either HOMA-IR, elevated waist circumference, or CRP after adjustment for covariates (Table 4). Instead, perceived neighborhood safety was associated with elevated waist circumference among women after adjustment for covariates (PR 1.06, 95% CI 1.02, 1.11). Increased physical activity was associated with a lower prevalence of insulin resistance and inflammation, and smoking was associated with a *lower* prevalence of insulin resistance among women. Among men, perceived neighborhood safety was associated with elevated HOMA-IR after adjustment for covariates (PR 1.25, 95% CI 1.08, 1.46), but not elevated waist circumference or inflammation. Neighborhood socioeconomic disadvantage was not associated with insulin resistance, elevated waist circumference or inflammation among men. Increased physical activity was associated with a lower prevalence of all three biomeasures among men (Table 4). Smoking was associated with a *lower* prevalence of insulin resistance among men, and a higher prevalence of elevated CRP among men.

**Table 3:** Correlates of Elevated Fasting Plasma Glucose in the Jackson Heart Study Prevalence Ratios and (95% Confidence Intervals).

	Adjusted for Age	Adjusted for Age and Health Behaviors^a^	Adjusted for All Covariates^b^
**Women** c			
**N = 2,454**			
Lives in most disadvantaged neighborhoods	1.18 (0.87, 1.60)	1.15 (0.86, 1.52)	1.14 (0.85, 1.52)
Perceives Neighborhood as Unsafe	1.36 (1.03, 1.80)	1.36 (1.03, 1.79)	1.36 (1.03, 1.80)
**Men**			
**N = 1,455**			
Lives in most disadvantaged neighborhoods	1.52 (0.94, 2.44)	1.44 (0.93, 2.24)	1.44 (0.93, 2.24)
Perceives Neighborhood as Unsafe	1.32 (1.03, 1.68)	1.31 (1.03, 1.66)	1.30 (1.02, 1.66)

Figures estimate associations with elevated fasting plasma glucose via binomial log regression with multiple imputations for missing covariates performed in PROC GENMOD, with repeated statement for neighborhood census tracts. Reference categories: Lives in most advantaged neighborhoods, does not perceives neighborhood as unsafe. Analysis for metabolic syndrome excludes all diabetics (self-reported, use of diabetic medications, elevated fasting plasma glucose equal or greater than 126 mg/dL, or hemoglobin A1C greater or equal to 6.5%) and individuals with ≤400 kcal daily energy intake.

a Models estimate the prevalence ratios associated with neighborhood socioeconomic disadvantage and perceived neighborhood safety adjusted for active living index, current smoking status, meeting USDA diet recommendations for percentage of calories as carbohydrate, fat, greater than 15% of daily calories as protein, daily dietary fiber intake, and percentages of calories as alcohol.

b Adjusts for age, health behaviors, lives in most disadvantaged neighborhoods, perceives neighborhood as unsafe, neighborhood stability, family household income scaled for family size, and less than high school educational attainment.

c Figures estimate the odds ratios associated with elevated fasting plasma glucose via binomial logit regression with multiple imputations for missing covariates performed in PROC GENMOD, with repeated statement for neighborhood census tracts.

**Table 4:** Biomeasures of Insulin Resistance, Central Adiposity, and Cardiovascular Inflammation among Women and Men in the Jackson Heart Study Prevalence Ratios and (95% Confidence Intervals).

	Women			Men		
	N = 2,454			N = 1,455		
	Elevated HOMA-IR	Elevated Waist Circumference	Elevated C-reactive Protein	Elevated HOMA-IR	Elevated Waist Circumference	Elevated C-reactive Protein
Lives in Most Disadvantaged Neighborhoods	0.97 (0.89, 1.06)	1.02 (0.97, 1.06)	1.05 (0.96, 1.15)	1.08 (0.94, 1.23)	0.97 (0.88, 1.07)	0.98 (0.84, 1.14)
Perceives Neighborhood as Unsafe	1.01 (0.92, 1.10)	1.06 (1.02, 1.11)	1.05 (0.98, 1.13)	1.25 (1.08, 1.46)	1.05 (0.93, 1.20)	0.93 (0.79, 1.10)
Active Living Index	0.90 (0.85, 0.95)	1.00 (0.97, 1.03)	0.89 (0.85, 0.94)	0.89 (0.81, 0.97)	0.92 (0.85, 1.00)	0.80 (0.72, 0.89)
Current smoker	0.85 (0.74, 0.98)	1.02 (0.95, 1.10)	1.08 (0.95, 1.21)	0.74 (0.58, 0.93)	1.01 (0.87, 1.18)	1.48 (1.28, 1.71)

Figures estimate associations with elevated insulin resistance (HOMA-IR) greater than or equal to 3.31, elevated waist circumference greater than 88 cm, and elevated C-reactive protein greater or equal to 3.0 mg/L. Prevalence ratios estimated via binomial log regression with multiple imputations for missing covariates performed in PROC GENMOD, with repeated statement for neighborhood census tracts. Analysis excludes all diabetics (self-reported, use of diabetic medications, elevated fasting plasma glucose equal or greater than126 mg/dL, or hemoglobin A1C greater or equal to 6.5%), individuals with ≤400 kcal daily energy intake and individuals with HOMA-IR <0. Analyses are fully adjusted for listed covariates plus age, neighborhood stability, dietary intake (meeting USDA recommendations regarding the percentage of calories in carbohydrate and fat consumed, exceeding 15% of calories as protein, dietary fiber intake, and percentage of calories consumed as alcohol), scaled family household income, and less than high school educational attainment. Reference categories: Lives in most advantaged neighborhoods, does not perceives neighborhood as unsafe, former or never smoker. Active Living Index modeled as a continuous variable.

## Discussion

Our study found that living in socioeconomically disadvantaged neighborhoods was associated with an increased prevalence of metabolic syndrome among African American women. Moreover, we found that a lack of perceived neighborhood safety was associated with an increased prevalence of elevated fasting glucose and elevated waist circumference among women, and with elevated fasting glucose and insulin resistance among men. Living a physically active lifestyle, some dietary habits, and smoking behaviors were associated with cardiometabolic risks. However, these behavioral characteristics did not reduce or attenuate the associations we observed with neighborhood socioeconomic disadvantage or lack of perceived neighborhood safety.

We observed these associations in specific social and biological contexts among JHS participants. The great majority of JHS participants lived in urban disadvantaged neighborhoods in the southeastern United States, an area characterized by impoverished neighborhoods, high unemployment, evidence of material deprivation (lack of car ownership), and where the lack of perceived safety was highly common. Additionally, at the time they were surveyed, JHS participants in this study were non-diabetic, but were at a stage in the life-course in which a vast majority already experienced metabolic derangements associated with aging and chronic stress, particularly abdominal adiposity and cardiovascular inflammation among women, and elevated blood pressure among men and women [37]. In this biosocial context, we found that derangements of glucose metabolism among men and women, insulin resistance among men, and abdominal adiposity among women were key physiologic disturbances specifically associated with neighborhood deprivation and perceived lack of safety. Our findings suggest that future research in African American populations in similar contexts should specifically track glucose metabolic pathways in interventions that focus on neighborhood or psychosocial stressors associated with neighborhood disadvantage and disorder.

Our findings are concordant with work in randomized controlled trial settings among urban African American women that found moving from high to low poverty neighborhoods was associated with reductions in extreme obesity and prevalent diabetes [38]. Our sex-specific findings of an association between the metabolic syndrome and neighborhood socioeconomic disadvantage among women but not men may relate, in part, to individual-level socioeconomic status differences by sex in the JHS, where women may have had fewer resources to buffer potential effects of low socioeconomic status neighborhoods compared to men. The finding of strong a relation between socioeconomic position and the metabolic syndrome among women, but not men has been observed in the literature [39], [40]. In the Monitoring Trends and Determinants in Cardiovascular Disease (MONICA) study, individual-level household income was associated with the metabolic syndrome among women but not men [40]. Similarly, Loucks et al. find a strong relation between individual-level socioeconomic position (SEP) measured by educational attainment, and the metabolic syndrome among women, but not among men, in data from the third National Health and Nutrition and Examination Survey (NHANES III) [39]. The authors of these studies posit that women with low SEP may have greater psychosocial risks for the metabolic syndrome, including depressive symptomatology [39], [40]. Additional studies that evaluate measures of psychosocial stress symptoms may be useful to further elucidate mechanisms that underlie sex differences in susceptibility to the metabolic syndrome associated with poor socioeconomic conditions. Unlike prior work in the Coronary Artery Risk Development in Young Adults (CARDIA) study among non-diabetic young African American and white men, we did not find that insulin resistance was associated with neighborhood disadvantage in the middle-aged men in the JHS [41]. Instead, we found that insulin resistance – at a level shown to predict future development of diabetes in a separate African American cohort [24] – was related to the psychosocial exposure of perceived neighborhood safety.

Our study adds to prior work by contributing rarely available data on specific physiologic mechanisms associated with disadvantage and disorder, particularly, measures of insulin resistance and inflammation among African Americans. Though each of these pathways is associated with HPA-axis stimulation, our study strengthens the hypothesis that glucose metabolism, and insulin resistance may be more closely tied to neighborhood disadvantage and disorder than other features of HPA-axis stimulation, namely dyslipidemia, as also seen in the Neighborhood Deprivation and Cardiometabolic Risk Factors in the Diabetes Study of Northern California (DISTANCE) study by Laraia et al. Our study also adds to prior work by including a measure of neighborhood socioeconomic disadvantage with strong reliability in neighborhoods in the southeastern United States, which showed convergent validity with other concepts rooted in the literature on Social Disorganization Theory [26]. Additionally, our study adds to the investigation of independent associations between neighborhood disadvantage, disorder and cardiometabolic risks by incorporating measures of dietary intake from a culturally appropriate food frequency questionnaire developed for use in this cohort.

Our data have important limitations that should be considered. First, the study design was cross-sectional and we cannot provide information on the causal relation between neighborhood disadvantage, disorder, and cardiometabolic risks. Our measure of perceived neighborhood safety, in particular, was assessed retrospectively and may have underestimated the strength of these associations compared to a concurrent assessment of perceived safety. Moreover, there were missing data on perceived neighborhood safety due to the timing of survey administration, and on income due to item non-response. We used multiple imputation techniques to avoid bias associated with list-wise deletion of these responses [33]. Additionally, we assessed relationships among neighborhood disadvantage, disorder, and cardiometabolic risks during middle and older age, and cannot assess the impact of early childhood or young adult exposures that may have been associated with the physiologic abnormalities that were already highly prevalent at baseline in the JHS. Last, we suggest that future work should also examine factors that confer resilience within neighborhoods and the ways in which countervailing forces such as neighborhood-level collective efficacy or wealth offer protective influences.

In summary, our work contributes to the literature by finding specific cardiometabolic risks factors that are independently associated with neighborhood disadvantage and disorder in middle-aged and older African American populations. Glucose metabolism and insulin resistance should be targeted and tracked in intervention studies designed to mitigate and eliminate material and psychosocial disadvantages associated with neighborhood contexts. Importantly, among JHS participants, more than 70% were exposed to disadvantaged neighborhoods, and a substantial percentage perceived that these neighborhoods were unsafe. Given the pervasive nature of these exposures, population-level interventions against neighborhood disadvantage and disorder have great potential to impact the biology of disadvantage that we observed, and should be considered as a strategy to achieve health equity for African American populations in high risk places.

## Supporting Information

Table S1 **Contextual Characteristics: Defining Neighborhood Socioeconomic Disadvantage in Jackson Heart Study Neighborhoods.** Figures represent ecological associations at the neighborhood (census tract) level. Kruskal-Wallis statistic used to test for non-parametric associations between categorical neighborhood disadvantage and continuous measures of related contextual characteristics. Related contextual characteristics are measures from the Theory of Social Disorganization.
(DOCX)
