# Supplementary material for: Neighborhood Disadvantage, Neighborhood Safety and Cardiometabolic Risk Factors in African Americans: Biosocial Associations in the Jackson Heart Study
Source: PLoS One. 2013 May 14;8(5):e63254. doi: 10.1371/journal.pone.0063254 (PMC3653956; doi:10.1371/journal.pone.0063254)
Supplement: Table S1 — Contextual Characteristics: Defining Neighborhood Socioeconomic Disadvantage in Jackson Heart Study Neighborhoods. Figures represent ecological associations at the neighborhood (census tract) level. Kruskal-Wallis statistic used to test for non-parametric associations between categorical neighborhood disadvantage and continuous measures of related contextual characteristics. Related contextual characteristics are measures from the Theory of Social Disorganization. (DOCX) [file pone.0063254.s001.docx]

**Table S1. Contextual Characteristics: Defining Neighborhood Socioeconomic Disadvantage in Jackson Heart Study Neighborhoods**

|  | Most Advantaged Neighborhoods | | Most Disadvantaged Neighborhoods | P Value |
| --- | --- | --- | --- | --- |
| Census 2000 Characteristics | N = 51 | | N = 51 |  |
| **Neighborhood Socioeconomic Disadvantage (NSED) Score, median (IQR)** | | 11 (6, 15) | 35 (27, 50) | - |
| **NSED components** |  | |  |  |
| Percent of adults below poverty, % | 7 | | 24 | - |
| Civilian unemployment rate, % | 3.6 | | 10.7 | - |
| Percent of households without a car, % | 3 | | 13 | - |
| **Convergent validity with related contextual characteristics** |  | |  |  |
| Percent college educated, % | 37 | | 13 | <0.0001 |
| Percent of housing that is owner occupied, % | 84 | | 57 | <0.0001 |
| Percent of neighbors in place since 1995, % | 52 | | 57 | 0.38 |
| Percent of population aged 15 to 24, % | 13 | | 17 | <0.0001 |
| Percent women listed as head of household, % | 11 | | 31 | <0.0001 |
| Percent black population, % | 18 | | 83 | <0.0001 |
| Percent of census tract in urban areas, % | 89 | | 100 | <0.001 |

Figures represent ecological associations at the neighborhood (census tract) level. Kruskal-Wallis statistic used to test for non-parametric associations between categorical neighborhood disadvantage and continuous measures of related contextual characteristics. Related contextual characteristics are measures from the Theory of Social Disorganization.
